# Pathogenic Microenvironment from Diabetic–Obese Visceral and Subcutaneous Adipocytes Activating Differentiation of Human Healthy Preadipocytes Increases Intracellular Fat, Effect of the Apocarotenoid Crocetin

**DOI:** 10.3390/nu13031032

**Published:** 2021-03-23

**Authors:** Lesgui Alviz, David Tebar-García, Raquel Lopez-Rosa, Eva M. Galan-Moya, Natalia Moratalla-López, Gonzalo L. Alonso, Eduardo Nava, Sílvia Llorens

**Affiliations:** 1 Seguro Social de Salud del Perú (EsSalud) Andahuaylas, Apurímac 03701, Peru; lesguialviznahui@gmail.com; 2 Translational Oncology Laboratory, Centro Regional de Investigaciones Biomédicas (CRIB), Universidad de Castilla-La Mancha, 02008 Albacete, Spain; David.Tebar@uclm.es (D.T.-G.); Raquel.LRosa@uclm.es (R.L.-R.); EvaMaria.Galan@uclm.es (E.M.G.-M.); 3 Cátedra de Química Agrícola, ETSI Agrónomos y de Montes, Universidad de Castilla-La Mancha, Campus Universitario, 02071 Albacete, Spain; Natalia.Moratalla@uclm.es (N.M.-L.); Gonzalo.Alonso@uclm.es (G.L.A.); 4 Department of Medical Sciences, Faculty of Medicine of Albacete, Centro Regional de Investigaciones Biomédicas (CRIB), University of Castilla-La Mancha, 02008 Albacete, Spain; eduardo.nava@uclm.es

**Keywords:** adipogenesis, diabesogenic microenvironment, crocetin

## Abstract

In diabetes mellitus type 2 (DM2), developed obesity is referred to as diabesity. Implementation of a healthy diet, such as the Mediterranean, prevents diabesity. Saffron is frequently used in this diet because of its bioactive components, such as crocetin (CCT), exhibit healthful properties. It is well known that obesity, defined as an excessive accumulation of fat, leads to cardiometabolic pathology through adiposopathy or hypertrophic growth of adipose tissue (AT).This is related to an impaired adipogenic process or death of adipocytes by obesogenic signals. We aimed to evaluate the effect of the pathogenic microenvironment and CCT, activating differentiation of healthy preadipocytes (PA). For this, we used human cryopreserved PA from visceral adipose tissue (VAT) and subcutaneous adipose tissue (SAT) depots obtained from healthy and obese-DM2 donors. We studied the effect of a metabolically detrimental (diabesogenic) environment, generated by obese-DM2 adipocytes from VAT (VdDM) or SAT (SdDM), on the viability and accumulation of intracellular fat of adipocytes differentiated from healthy PA, in the presence or absence of CCT (1 or 10 μM). Intracellular fat was quantified by Oil Red O staining. Cytotoxicity was measured using the MTT assay. Our results showed that diabesogenic conditions induce cytotoxicity and provide a proadipogenic environment only for visceral PA. CCT at 10 μM acted as an antiadipogenic and cytoprotective compound.

## 1. Introduction

Type 2 diabetes mellitus (DM2) is a metabolic disorder mainly characterized by insulin resistance (IR) and abnormal insulin secretion by pancreatic cells β [1]. It is currently considered a major public health problem with significant social and economic impacts. DM2 represents 90% of the total cases of DM and it is on the list of the ten leading causes of death [2]. Obesity and DM2 have such a strong relationship that obesity developed in DM2 is referred to as diabesity [3]. 

The World Health Organization defines obesity as an abnormal or excessive accumulation of fat (adiposity) that can be a risk factor for suffering DM2, cardiovascular disease, or cancer [4,5]. Paradoxically, there are both metabolically healthy obese individuals and lean metabolically obese subjects [6], as well as obese and non-obese DM2 patients [7]. It appears that the pathogenesis of obesity is not just a matter of fat quantity, but also of adipose tissue (AT) dysfunction, which is known as adiposopathy [4]. 

Adiposopathy is characterized by pathologic AT growth in response to permanent positive energy uptake. The growth of AT takes place by hypertrophy (by increasing the size of existing adipocytes) and hyperplasia (by increasing the number of adipocytes, generating adipocytes de novo) [8]. The latter is produced by means of the adipogenesis process, which is responsible for maintaining a constant mass of adipocytes in adult individuals through a delicate balance between apoptosis and adipogenesis. This process leads to the production of new adipocytes from preadipocytes (PA) [9], inducing (via molecular signaling) morphological and functional differentiation of PA to an adipocytic phenotype. Since mature adipocytes are post-mitotic, the expansion by hyperplasia derives from the activation of PA [8]. Adiposopathy or hypertrophic AT growth is generated by a reduced capacity to expand and accommodate the surplus of energy [10]. If this capacity is exceeded, adipocytes degenerate and die by pyroptosis [11], leading to ectopic fat accumulation, systemic inflammation, IR, and development of DM2 [12,13]. Two AT depots are involved: visceral adipose tissue (VAT) and subcutaneous adipose tissue (SAT)—the latter despite frequently being considered as non-pathogenic [4,14]. The type of expansion and the secretory profile of adipocytes within both depots are key research points. Under physiological conditions, SAT acts as a physiological buffer of the excessive nutrient intake. To adjust the oversupply, the adipogenic process is activated, leading to hyperplasia, which increases the capacity to store more lipids and helps maintain a stable metabolic state in the individual [14]. Dysregulation of the fat stores carries harmful implications for metabolic health and generates pathogenic microenvironments that influence the type of expansion of each depot. Growth of these depots and the mechanisms that regulate it is fundamental for the development of obesity and its associated comorbidities. While the role of adiposopathy in the pathological response to positive caloric balance is well established, the mechanisms that drive an impaired activation of adipogenesis or its pathologic role, independently of an increased demand for fat storage, is still unclear. One hypothesis suggests that the PA of the different depots have different intrinsic abilities to respond to obesogenic signals [15,16]. Some studies propose that mechanisms specifically inherent of the PA are responsible for the particular differences that each depot shows in terms of gene expression, potential for in vitro differentiation, and the contribution to metabolic diseases [17,18]. In contrast, Jeffery et al., suggest that the proliferation and differentiation of PA within each depot are determined by the microenvironment surrounding the depot rather than any intrinsic property of PA [19]. 

A greater increase in the accumulation of lipids within the adipocyte, due to an impaired adipogenic process triggered by obesogenic signals, is a key factor in obesity [20]. Therefore, a possible therapy to prevent the development of adipose tissue and obesity focuses on the regulation of adipogenesis in relation to the reduction of fat mass [21]. The implementation of a healthy diet is part of the initial treatment of DM2 to prevent the development of obesity and to modify risk factors, such as hyperlipidemia and hypertension. The Mediterranean diet is an example of a healthy diet, which is associated with a reduction in the risk of cardiovascular disease, the incidence of obesity, and lower mortality from cardiometabolic diseases and cancers [22]. Saffron is a component frequently used in this diet [23,24]. Plants classified as anti-obesity in functional diets usually present a high antioxidant activity. Our present work is focused on the crocetin (CCT) derived from saffron (*Crocus sativus* L.). CCT is an apocarotenoid with a high antioxidant power, which has demonstrated multiple pharmacological properties, such as anti-inflammatory, antiatherosclerotic, and anticarcinogenic [25]. Moreover, it has been shown that CCT can prevent the accumulation of visceral fat and IR induced by a hypercaloric diet in rats, without altering feed consumption [26].

In this work, we aimed to evaluate if the differentiation of new adipocytes within each depot is impaired when it is activated by a potential pathogenic microenvironment, and if CCT has any ability to restore this possible impairment. For this, we used the secretomes generated by the differentiated adipocytes from VAT and SAT depots of obese DM2 patients to simulate the pathogenic microenvironments, which we named “diabesogenic” because they would display both diabetogenic and obesogenic signals. We studied the effect of these pathological microenvironments on the viability and accumulation of intracellular fat of adipocytes differentiated from PA obtained from VAT and SAT depots of healthy, non-obese individuals, in the presence or absence of CCT.

## 2. Materials and Methods

### 2.1. Plant Material and Isolation of CCT

Saffron was obtained from the “Agrícola Técnica de Manipulación y Comercialización” company (Minaya, Albacete, Spain) during the 2014–2015 harvest. Dried stigmas belonged to the Protected Designation of Origin (PDO) “Azafrán de La Mancha”, which guarantees their origin and freedom from possible fraud. This saffron complies with the ISO 3632:2011 standard, and it is classified as Category I. Very low moisture saffron specimens were stored in the dark at 4 °C until further use. 

CCT was obtained by hydrolysis of aqueous solutions of the saffron sample using a protected internal method of “Verdú Cantó Saffron Spain” company (Novelda, Alicante, Spain) [27]. CCT was analyzed by the reversed phase high-performance liquid chromatography–diode array detection (RP–HPLC–DAD) technique, as we previously reported [28]. A total of 20 µL of aqueous extracts of CCT were injected into an Agilent 1200 chromatograph (Palo Alto, CA, USA), previously filtered through a filter made of hydrophilic polytetrafluoroethylene (PTFE) with a pore size of 0.45-µm (Millipore, Bedford, MA, USA). The chromatographic determination was achieved by using a 150 × 4.6 mm, 5 μm (inner diameter), Luna C18 column (Phenomenex; Le Pecq Cedex, Luna, France) that was equilibrated at 30 °C. The eluents were acetonitrile (ACN) and Milli Q water, which were used as mobile phase at flow rate of 0.8 mL/min. HPLC-grade ACN was obtained from Panreac^®^ (Barcelona, Spain) and ultrahigh-purity water was produced using a Milli-Q system (Millipore, Bedford, MA, USA). The elution gradient was set up for solvent ACN as follows: 20%, 0–5 min; 20–80%, 5–15 min; 80%, 15–18 min; and 20%, 18–30 min. The DAD detector (Hewlett Packard, Waldbronn, Germany) was set at 440 nm for cis/trans CCT detection. Chromatographic purity at 440 nm of cis/trans-CCT was 99%, being 86% the proportion of trans-CCT (retention time: 16.64 min) and 13% of cis-CCT (retention time: 17.86 min). CCT was stored at −20 °C until further use, obtaining again the same chromatographic purity before it was used. 

### 2.2. Cell Lines and Cell Culture

Human visceral and subcutaneous PA from healthy and DM2-obese donors, were purchased at Lonza (Basel, Switzerland), as Cryopreserved Poietics™. Briefly, healthy PA, visceral (HV) and subcutaneous (HS), were isolated from kidney and bladder VAT and SAT, respectively. They were obtained from non-diabetic and non-obese donors. According to the provided information, diabetic/obese PA (DM2–PA) were isolated from VAT and SAT, obtained from a 76-year-old obese patient with DM2. 

Cells were cultured, maintained, and differentiated according to the manufacturer’s instructions, with slight modifications. In all experiments, cells were used within the second passage. Briefly, PA were expanded, until reaching an optimal confluence (70–80%), in a 75 cm2 flask at 37 °C under a humidified 5% CO2 atmosphere in a PA growth medium (PGM2, PGM-2TM Preadipocyte Growth Medium-2 BulletKit^TM^ from Lonza). This medium contains PA basal medium (PBM2, 90%), supplemented with L-glutamine (GLUT, 1%), gentamicin (30 µg/mL) and amphotericin (15 ng/mL), and inactivated fetal bovine serum (FBS, 10%). Confluents cells were seeded in sterile well plates. In the case of healthy PA, in 96-well plates (P96) at a cell density of 1 × 10^4^ cells/well. In the case of DM2–PA, in 6-well plates (P6) at a cellular density of 3 × 10^4^ cells/well. Previously, cells were counted in an automatic counter (TC10 Automated Cell Counter, Hercules, CA, USA, Bio-Rad). For this, the dye exclusion test Trypan Blue (TB, 0.4%) was used. Only cells with intact membranes can effectively exclude the dye, dead cells become stained [29]. 

#### Induction of the Differentiation Process

After optimal confluence in plate (48–72 h), the adipogenic process was stimulated by adding adipogenic differentiation medium (ADM), which contained PGM2 plus an adipogenic induction cocktail containing: 3-isobutyl-1-methylxanthine (IBMX), dexamethasone (DEX), insulin (INS), and indomethacin (IND), acquired from Lonza.

DM2–PA were maintained with ADM for 17 days. On the last day, secretomes obtained from differentiated visceral and subcutaneous DM2–PA, (referred to as “VdDM” and “SdDM”, respectively), were collected in aliquots (500μL) and immediately frozen at −80 °C, until further use. The same day that these secretomes were collected, differentiation of healthy PA (HV and HS) was set to start (conditions, controls, and media used are shown in Table 1). The differentiation of healthy PA was induced by adding ADM, VdDM, or SdDM, in the presence or absence of CCT (yielding final concentrations of 1 or 10 μM in the well). The choice of these particular concentrations is based on our experience in previous studies [27]. CCT concentrations were prepared from a stock CCT solution dissolved in sterile dimethyl-sulfoxide (DMSO), and added to ADM, VdDM, or SdDM (yielding a final concentration of DMSO in well, which was 0.001% (*v*/*v*). The differentiation of healthy PA was also carried out in the presence of sterile DMSO (0.001%), as the solvent control. PA were differentiated by adding ADM, VdDM, or SdDM, with or without CCT, for 17 days. Undifferentiated healthy PA (UD) were maintained with PGM2 also for 17 days (negative control of differentiation). On day 17, cellular viability and intracellular fat were determined.

### 2.3. Determination of Cell Viability: MTT Assay

Cell viability, measured as cellular metabolic activity, was determined using a colorimetric assay based on the metabolic reduction of 3-(4,5-dimethylthiazol-2-yl)-2,5-diphenyltetrazolium bromide (MTT) to formazan, mediated by the mitochondrial enzyme oxidoreductase succinate dehydrogenase; thus, reflecting the mitochondrial activity of the cells, and consequently the number of viable cells present. This assay is an indirect estimation of cell mass [30]. Formazan was solubilized by the addition of DMSO, resulting in a violet colored solution. The number of living cells is proportional to the amount of formazan produced. Seventeen days after induction of differentiation, the medium was aspirated and the cells were washed with Dulbecco’s Modified Eagle Medium (DMEM), with neither phenol red nor FBS (to avoid the interference of phenol red in the colorimetric assay). Next, MTT solution prepared freshly in sterile phosphate buffered saline (PBS), (0.5 mg/mL, 100 µL/well), was added and incubated for 2 h at 37 °C, in darkness. Then, MTT was replaced by the same volume of DMSO, and gently stirred to each well for 3–5 min. Finally, absorbance was determined by spectrophotometry at 595 nm (ASYS UVM 340, Cambridge, UK, Microplate Readers) [31]. The maximum absorbance detected for control differentiation (ADM) was set at 100% cellular viability and relative viability of differentiated adipocytes in the different diabesogenic microenvironments in the absence or presence of CCT. 

### 2.4. Determination of Intracellular Fat: Staining O Red Oil

Adipocytes were stained with O Red Oil (ORO), according to previous studies [32,33]. ORO is a lipophilic dye that specifically stains the triglycerides stored within the adipocytes. Previously, a concentrated solution of ORO (0.4 g/200 mL isopropanol) was prepared and continuously stirred for 24 h, at room temperature in darkness. It was filtered and stirred for another 24 h in the same conditions. The dye solution was prepared freshly in bi-distilled water (60:40 (*v*/*v*)). The medium was removed, and the adipocytes were washed 3 times with PBS, fixed with 4% formaldehyde (50 µL each well), and incubated for 1 h, at 37 °C, in darkness. Next, cells were washed with cold PBS, and dried for 10 min. Then, cells were incubated for 10 min with ORO (50 µL each well). After, the cells were washed three times with PBS, and dried for 15 min. Finally, isopropanol (50 µL each well) was added. Absorbance was measured at 517 nm with the aforementioned spectrophotometer. The maximum absorbance detected for control differentiation (ADM) was set at 100% intracellular fat and relative intracellular fat at differentiated adipocytes by diabesogenic microenvironments, with or without, CCT. 

Reagents, if not specified otherwise, have been acquired from Sigma-Aldrich.

### 2.5. Quantitative RT-PCR

RNA isolation of all samples was performed using a DANAGENE Tissue/Cells RNA Kit (Danagen-Bioted, S.L., Barcelona, Spain), following manufacturer instructions. RNA concentration and purity were determined using a NanoDrop ND-1000 spectrophotometer (Thermo Fisher Scientific Inc., Waltham, MA, USA.). Next, 1 μg of total RNA was reverse-transcribed using a RevertAid H Minus First Strand cDNA synthesis kit (Thermo Fisher Scientific Inc., Waltham, MA, USA) in a thermocycler (Bio-Rad) under the following reaction conditions: 65 °C for 5 min, 42 °C for 60 min, and 70 °C for 10 min. The resulting cDNAs were used to perform quantitative real-time PCR (qRT-PCR) analysis using a Fast SYBR Green Master Mix (Thermo Fisher Scientific Inc., Waltham, MA, USA) in a StepOnePlus Real-Time PCR system (Applied Biosystems, Thermo Fisher Scientific Inc., Foster City, CA, USA). The conditions used included an initial step at 95 °C for 10 min, followed by 40 cycles at 95 °C for 15 s and a final step at 60 °C for 1 min. Each sample was analyzed per triplicate, and cycle threshold (Ct) values of transcripts were determined using StepOne Software v.2.1. Ct values were normalized using 18S as reference. Healthy PA differentiated were used as controls to determine the relative fold-changes in messenger RNA (mRNA) expression, using the method explained by Livak and Schmittgen [34]. Primer sequences are listed in the following Table 2.

### 2.6. Determination of Adipokines in the Secretomes

**Figure 1:**
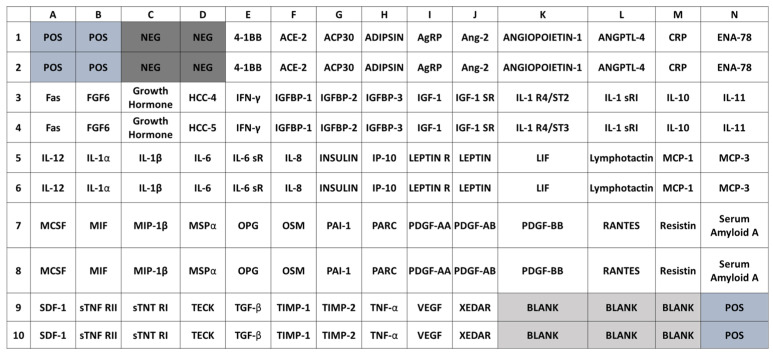
Spot map of anti-adipokine antibodies. Captured antibodies are spotted on a membrane with each pair of spots representing a different adipokine. Positive (POS), negative (NEG), and BLANK control spots are highlighted. POS spots: printed with biotin-conjugated Immunoglobulin (Ig) G protein; NEG spots: printed with the same buffer used to dilute antibodies printed on the array; BLANK spots: no printing.

Adipokines secreted from DM2–PA after their differentiation were detected using a human obesity antibody array (Abcam, Cambridge, UK, Reino Unido). For this purpose, we used 1 mL secretome collected on the last day of the differentiation process. For each secretome, a membrane capable of detecting 62 adipokines was used (Figure 1). For the reference array, a 1-mL aliquot of ADM, which had not been in contact with the PA, was used.

The assay was performed as follows: first, membranes were blocked with 2 mL blocking buffer and kept at room temperature for 30 min. Subsequently, buffer was aspirated, 1 mL of undiluted secretomes (SdDM or VdDM) were added and the membranes incubated at 4 °C overnight. The following day, the samples were aspirated again and the membranes washed following the manufacturer’s instructions. Subsequently, 1 mL of the biotin-conjugated anti-adipokine antibody cocktail was added to each membrane, and incubated at 4 °C overnight. After this, the solution was aspirated, the washing procedure repeated and 2 mL of horseradish peroxidase (HRP)-conjugated streptavidin was added to each membrane and incubated at 4 °C overnight. After carrying out once again the washing process, the membranes were transferred to blotting paper. For image developing, a mixture of equal volumes of two detection buffers (provided by the manufacturer) was added and kept for 2 min at room temperature. Images were obtained with a charge-coupled device (CCD). The membranes were exposed accordingly to obtain a low background and strong signal image from the positive controls.

Comparisons between the densities of the adipokine spots were made using the data obtained by densitometry by the ImageJ software program. For each spot, a circle of the same size was made and the total density per unit area was determined. The mean of negative control spots (NEG) was subtracted for background correction, and the densities normalized to the positive control spots (POS) signals on each membrane.

### 2.7. Statistical Analysis

The results are presented as the mean ± SEM of several repetitions of each condition. One-way ANOVA and (post-test) Bonferroni Multiple Comparison Test, using GraphPad Prism version 5.0 software, were used for differences between groups. The results were considered to be significant when the value of *p* was <0.05. The following comparisons were made: comparisons between visceral and subcutaneous adipocytes exposed to the same microenvironment, and between the same types of adipocyte exposed to different microenvironments. Comparisons of mRNA expression were determined by Student’s t-test (two-tailed). Differences of semi-quantitative data of adipokines from arrays were calculated using Mann–Whitney test. Values of *p* less than 0.05 were considered to indicate a significant statistically difference.

## 3. Results

### 3.1. Induction of the Differentiation Process of Healthy PA with an SdDM Secretome: Effect of CCT

#### 3.1.1. Effect on Cell Viability

CCT reverted the decrease in viability of visceral adipocytes during differentiation with SdDM.

**Figure 2:**
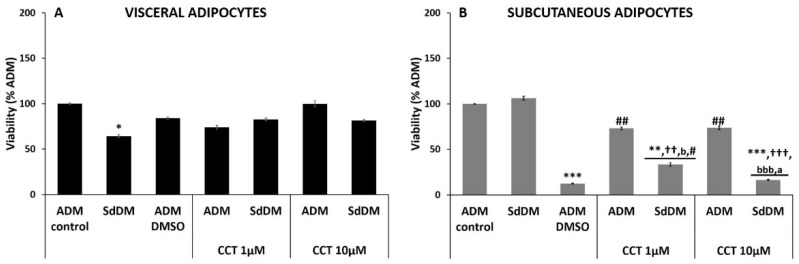
Effect of the SdDM secretome and CCT on the viability of adipocytes. The maximum cell viability detected for control differentiation (ADM-control) was set at 100%. Relative values of adipocyte viability in different conditions are shown: SdDM-differentiated cells, ADM–DMSO, and CCT applied to ADM- and SdDM-differentiated cells. Values were obtained at 17 days post-induction. Panel (**A**): percentage of cellular viability of visceral adipocytes. Panel (**B**): percentage of cellular viability of subcutaneous adipocytes. Bars indicate mean ± SEM of absorbance (λ595 nm) from at least three replicates of each condition. Significant differences are represented as: *, ** *** compared to ADM-control *p* < 0.05, *p* < 0.01, and *p* < 0.001, respectively. ††,††† compared to SdDM-control *p* < 0.01, and *p* < 0.001, respectively. #, ## compared to ADM–DMSO, *p* < 0.05 and *p* < 0.01, respectively. “a comparisons between CCT doses, *p* < 0.05. “b, bbb” comparison between SdDM–CCT and ADM–CCT (same dose), *p* < 0.05 and *p* < 0.001, respectively.

HV PA differentiated with SdDM showed a significant decrease in their viability (64.3 ± 1.7%; *p* < 0.05) (Figure 2, panel A and Table 3), compared to viability in ADM-healthy differentiated cells. However, the presence of CCT during the differentiation induced by SdDM, restored viability: CCT 1 µM up to 82.7 ± 1.5%, and CCT 10 µM up to 81.6 ± 1.2%. This result suggests that CCT exerts a protective role on the viability of visceral adipocytes when they are differentiated with SdDM. The percentage of viability obtained by HV PA differentiated with ADM or SdDM in the presence of CCT 1 µM or 10 μM was not significantly different to that obtained without CCT and this viability was not different between each other. The control solvent (ADM–DMSO) did not significantly affect the viability (Table 3).

Neither SdDM nor CCT affected viability during differentiation of subcutaneous adipocytes, but CCT protected against DMSO cytotoxicity

The induction of differentiation of HS PA with SdDM did not produce significant differences in cell viability (106.3 ± 2.1%) as compared to the induction of differentiation of these cells with ADM-control (Figure 2, panel B and Table 3). None of the CCT concentrations affected cell viability as compared to the same ADM-differentiated cells, although there was a trend to decrease (73.2 ± 1.5%, 73.7 ± 2.0% with 1 or 10 µM CCT, respectively), but did not reach statistical significance. These ADM–CCT viability values were significantly higher than the viability obtained by differentiating using ADM–DMSO, which drastically reduced the viability of subcutaneous adipocytes (12.4 ± 0.4%, *p* < 0.01), unlike that of visceral adipocytes. The effect on viability of either concentration of CCT was not different between each other. These data indicate that: 1) HS PA are extremely sensitive to the cytotoxic effect of DMSO, since the volume used (0.001%) rarely presents cytotoxicity [35], and 2) CCT exerts a protective role because it reverted DMSO-induced cytotoxicity of ADM-differentiated cells. In contrast, CCT failed to protect SdDM-differentiated cells: a significantly lower cell viability was measured (33.4 ± 1.8% and 16.4 ± 2.0%; *p* < 0.001, for CCT 1 and 10 μM, respectively) as compared with ADM-differentiated cells and significantly lower than SdDM-differentiated cells. This suggests that the combination of DMSO and SdDM is probably responsible for this decrease in viability despite the presence of CCT. Significant differences were also observed in the percentage of viability between the two concentrations of CCT added to SdDM-differentiated cells (Figure 2, panel B and Table 3, *p* < 0.05). It was also observed that the viability obtained in SdDM-differentiated cells treated with CCT 1 μM was significantly higher (*p* < 0.05) (Figure 2, panel B and Table 3) than that of cells incubated only with DMSO. This could be related to the fact that for the preparation of CCT 1 μM, a lower volume of DMSO is used, and therefore a lower toxicity is generated when compared with that of CCT 10 μM. The viability obtained with this latter concentration was not significantly different from that produced by DMSO alone.

#### 3.1.2. Effect on Cell Differentiation

CCT prevented the increase in intracellular fat of SdDM-differentiated visceral adipocytes.

**Figure 3:**
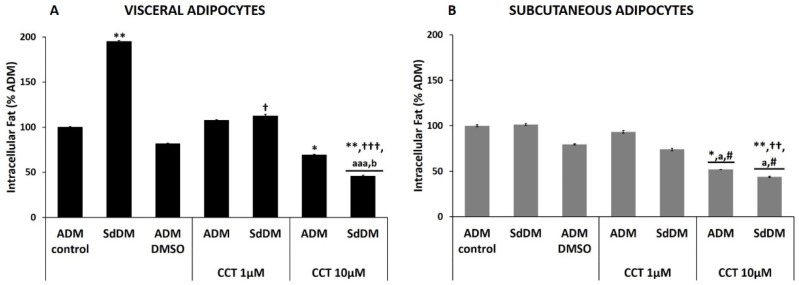
Effect of the SdDM secretome and CCT on the differentiation of healthy PA. The maximum intracellular fat detected for control differentiation (ADM-control) was set at 100%. Relative values of intracellular fat in different conditions are shown: SdDM-differentiated cells, ADM–DMSO, and CCT applied to ADM- and SdDM-differentiated cells. Values were obtained at 17 days post-induction. Panel (**A**): percentage of intracellular fat of visceral adipocytes. Panel (**B**): percentage of intracellular fat of subcutaneous adipocytes. Bars indicate mean ± SEM of absorbance (λ517 nm) from at least three replicates of each condition. Significant differences are represented as: *, ** compared to ADM-control *p* < 0.05 and *p* < 0.01, respectively. †, ††, ††† compared to SdDM *p* < 0.05, *p* < 0.01, and *p* < 0.001, respectively. # compared to ADM–DMSO, *p* < 0.05. “a, aaa” comparisons between CCT doses, *p* < 0.05 and *p* < 0.001, respectively. “b” comparison between SdDM–CCT and ADM–CCT (same dose), *p* < 0.05.

The SdDM secretome produced a significant increase of intracellular fat during the differentiation of HV PA (195.0 ± 1.0%; *p* < 0.01) as compared to the ADM-control (Figure 3, panel A, Table 4). No alteration in the accumulation of intracellular fat was observed with ADM–DMSO (81.7 ± 0.7%, Figure 3, panel A, Table 4). In ADM-differentiated cells, the presence of CCT 1 µM failed to exhibit any change in the percentage of their intracellular fat (107.5 ± 0.9%). In contrast, this dose of CCT significantly decreased intracellular fat levels in SdDM-differentiated cells (112.3 ± 2.0%; *p* < 0.05). These results indicate that, at this concentration, CCT did not exhibit antiadipogenic effect in physiological conditions, but in the presence of a pathological microenvironment, such as SdDM, CCT restored the adipogenic process of the adipocytes. When ADM-differentiated cells were treated with CCT 10 µM, a significant decrease in intracellular fat was observed (69.4 ± 0.6%; *p* < 0.05). In the case of SdDM-differentiated, the presence of CCT 10 µM elicited a marked decrease in intracellular fat 45.7 ± 1.1%, statistically different compared to SdDM-control (*p* < 0.001). These results indicate that SdDM generates a proadipogenic microenvironment for HV PA and that CCT 1 and 10 µM prevents the increase in intracellular fat to different extents. Furthermore, the highest dose of CCT also exhibits antiadipogenic effects, even on differentiation in a healthy environment.

High dose CCT inhibited the ADM and SdDM differentiation processes of subcutaneous adipocytes.

The differentiation of HS PA with SdDM did not affect the percentage of intracellular fat (101.3 ± 1.1%) compared to those differentiated with ADM-control (Figure 3, panel B and Table 4). This indicates that the diabesogenic microenvironment generated by the SdDM does not alter the adipogenic process of the HS PA. The percentage of intracellular fat obtained in the presence of DMSO (79.6 ± 0.7%) was not statistically different from that of ADM-differentiated cells. CCT 1 µM (93.3 ± 1.5%) did not change intracellular fat percentage neither to the ADM- nor the SdDM-differentiated cells (74.1 ± 1.4%). In contrast, CCT 10 µM significantly decreased intracellular fat (52.0 ± 0.2%; *p* < 0.05) of ADM-differentiated cells, indicating that this concentration of CCT inhibits the adipogenic process of these adipocytes. This decrease in intracellular fat was also statistically significant to that elicited by ADM–DMSO differentiation, indicating that the antiadipogenic effect is produced only by CCT. Likewise, this dose of CCT also produced a significant decrease in intracellular fat of SdDM-differentiated cells (43.9 ± 0.8%; *p* < 0.01) compared to the same cells in the absence of CCT (SdDM-control) but was not different from that observed in ADM–CCT 10 µM differentiated cells. These results indicate that SdDM does not affect the adipogenic process of the HS PA and that CCT at 10 µM acts as an antiadipogenic compound.

### 3.2. Induction of the Differentiation Process of Healthy PA with a VdDM Secretome: Effects of CCT

The results obtained for the control differentiation induced by ADM, DMSO, and CCT 1 and 10 µM are included in the previous Section 3.1.1 and Section 3.1.2.

#### 3.2.1. Effect on Cell Viability

High dose CCT restored viability of VdDM-differentiated visceral adipocytes.

**Figure 4:**
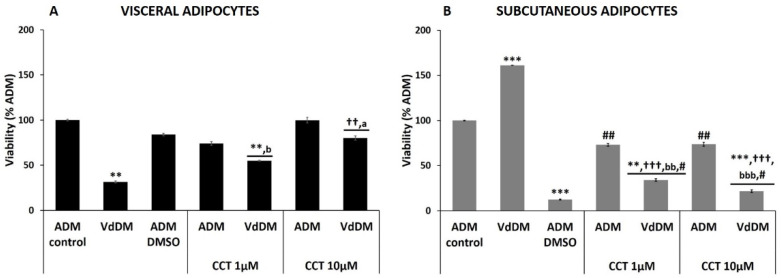
Effect of VdDM secretome and CCT on viability of adipocytes. The maximum cell viability detected for control differentiation (ADM-control) was set at 100%. Relative values of adipocyte viability in different conditions are shown: VdDM-differentiated cells, ADM–DMSO, and CCT applied to ADM- and VdDM-differentiated cells. Values were obtained at 17 days post-induction. Panel (**A**): percentage of cellular viability of visceral adipocytes. Panel (**B**): percentage of cellular viability of subcutaneous adipocytes. Bars indicate mean ± SEM of absorbance (λ595 nm) from at least three replicates of each condition. Significant differences are represented as: ** *** compared to ADM-control, *p* < 0.01 and *p* < *0*.001, respectively. ††, ††† compared to VdDM *p* < 0.01 and *p* < 0.001, respectively. #, ## compared to ADM–DMSO, *p* < 0.05 and *p* < 0.01, respectively. “a” comparisons between CCT dose, *p* < 0.05. “b, bb, bbb” comparison between VdDM–CCT and ADM–CCT (same dose), *p* < 0.05, *p* < 0.01, and *p* < 0.001, respectively.

The induction of differentiation of HV adipocytes with VdDM caused a significant decrease in viability (31.6 ± 1.0%; *p* < 0.01) compared to ADM-control, which was restored in the presence of CCT, although significance was reached only the highest dose (Figure 4, panel A and Table 3). The presence of CCT 1 µM in VdDM-differentiated adipocytes significantly decreased the cell viability (55.1 ± 0.4%; *p* < 0.01, compared to ADM-control and *p* < 0.05, compared to ADM–CCT 1 µM). However, the presence of CCT 10 µM in the VdDM significantly increased the viability up to 80.2% ± 2.4 (*p* < 0.01, compared to VdDM, and *p* > 0.05, compared to ADM–CCT 10 µM). Therefore, VdDM secretome is responsible for the decrease in cellular viability and CCT 10 µM exerts a protective role in restoring the viability.

VdDM increased cell viability during the differentiation process of subcutaneous adipocytes.

Unlike the differentiation of visceral adipocytes, in the case of the induction of differentiation of HS PA with VdDM (Figure 4, panel B and Table 3), a marked increase in viability was detected (161.3 ± 0.1%; *p* < 0.001, as compared with ADM-differentiated cells). This result contrasts with that obtained in the presence of SdDM, indicating that HS PA or mature subcutaneous adipocytes, are very sensitive to the diabesogenic environment provided by visceral adipocytes of DM2 origin. However, the presence of CCT in VdDM-differentiated adipocytes reduced the cell viability down to 34.1 ± 1.5% for CCT 1 µM and to 21.8 ± 3.3% for CCT 10 µM. This was similar to what occurred with the SdDM secretome. These results of viability were still higher than those produced by DMSO per se (ADM–DMSO, Table 3), indicating that CCT partially prevented the high toxicity exerted by DMSO to these adipocytes.

#### 3.2.2. Effect on Cell Differentiation

High dose CCT prevented the increase in intracellular fat of VdDM-differentiated visceral adipocytes.

**Figure 5:**
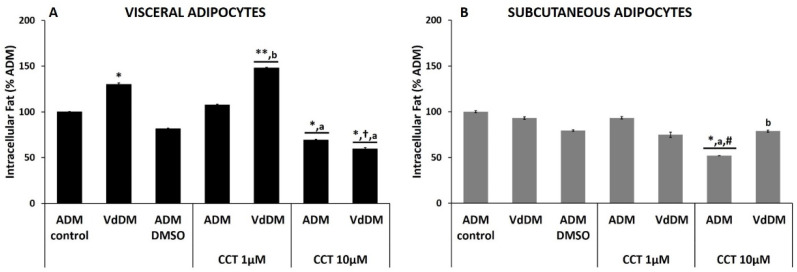
Effect of VdDM secretome and the CCT on the differentiation of healthy PA. The maximum intracellular fat detected for control differentiation (ADM-control) was set at 100%. Relative values of intracellular fat in different conditions are shown: VdDM-differentiated cells, ADM–DMSO, and CCT applied to ADM- and VdDM-differentiated cells. Values were obtained at 17 days post-induction. Panel (**A**): percentage of intracellular fat of visceral adipocytes. Panel (**B**): percentage of intracellular fat of subcutaneous adipocytes. Bars indicate mean ± SEM of absorbance (λ517 nm) from at least three replicates of each condition. Significant differences are represented as: *, ** compared to ADM-control *p* < 0.05 and *p* < 0.01, respectively. † compared to VdDM *p* < 0.05. # compared to ADM–DMSO, *p* < 0.05. “a” comparisons between CCT dose, *p* < 0.05. “b” comparison between VdDM–CCT and ADM–CCT (same dose), *p* < 0.05.

The activation of the differentiation of HV PA by incubating with the VdDM secretome produced a significant increase in intracellular fat (130.1 ± 1.5%; *p* < 0.05) compared to ADM-control (Figure 5, panel A and Table 4). This result indicates that, such as SdDM secretome, VdDM elicits proadipogenic effects during differentiation of HV PA. The presence of CCT 1 µM during differentiation with VdDM failed to elicit changes in intracellular fat (147.9 ± 0.7%) as compared with VdDM-control, although it was significantly enhanced compared to ADM-control (*p* < 0.01) or with its own ADM–CCT control (*p* < 0.05). In contrast to the low dose, CCT 10 µM present in VdDM-differentiated PA, showed a significant decrease in intracellular fat (59.4 ± 1.5%) as compared to VdDM and also to ADM-control (both, *p* < 0.05). Thus, CCT exhibits antiadipogenic abilities on both VdDM and non-diabesogenic environments (ADM-control).

Differentiation process of subcutaneous adipocytes was unaffected by VdDM secretome.

VdDM-differentiated subcutaneous adipocytes did not exhibit variations in the percentage of intracellular fat (93.1 ± 1.3%) as compared to ADM-control (Figure 5, panel B and Table 4). This indicates that the adipogenic process of the subcutaneous adipocytes is resistant to the diabesogenic microenvironment generated by visceral diabetic adipocytes. A tendency to lower intracellular fat of VdDM-differentiated cells was elicited by CCT 1 μM (74.9 ± 2.8%, *p* > 0.05 compared to ADM-control and ADM–CCT), but did not reach significance. On the other hand, treatment with CCT 10 μM in VdDM-differentiated adipocytes, unlike what observed in healthy visceral adipocytes, significantly increased intracellular fat (78.9% ± 1.1, *p* < 0.05) compared to his own control, ADM–CCT).

**Figure 6:**
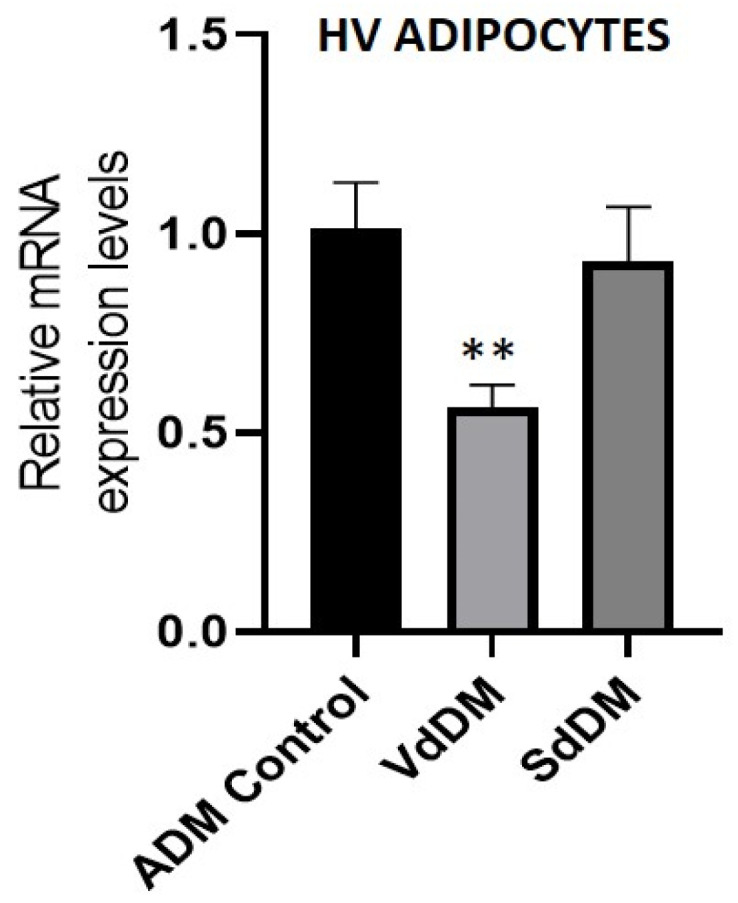
Quantification of PPAR-γ mRNA levels in HV PA differentiated in the presence of VdDM and SdDM. The maximum expression detected for control differentiation (ADM-control) was set at 1. Bars indicate mean ± SEM of relative expression from at least three replicates of each condition. Significant differences are represented as: ** compared to ADM-control, *p* < 0.01.

Overall, these results suggest that the differentiation of SAT and VAT-derived adipocytes with SAT or VAT-derived secretomes, leads to certain patterns of hypertrophy/hyperplasia. In agreement with this, evaluation of PPAR-γ gene expression, a marker of adipogenesis, was diminished in response to VdDM in comparison to ADM control and SdDM, contrary to what was initially expected (Figure 6). However, this decreased expression is in line with a pattern of hypertrophy in response to this particular secretome [36].

**Figure 7:**
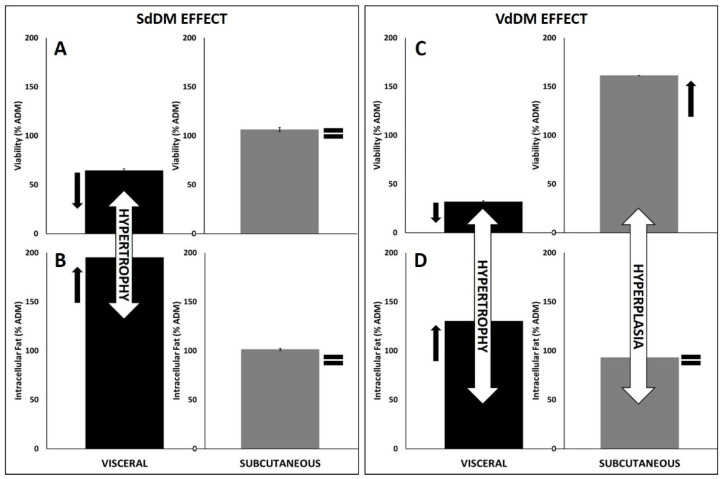
Schematic representation of the pattern effect of secretomes, as seen by juxtaposing viability and intracellular fat of adipocytes. Panel (**A**,**B**): effect of SdDM on viability and intracellular fat of visceral (left) and subcutaneous (right) adipocytes. Panel (**C**,**D**): effect of VdDM on viability and intracellular fat of visceral (left) and subcutaneous (right) adipocytes. The “=“ indicates no effect. Black arrows approaching indicate a tendency towards hypertrophic growth. Black arrows moving away indicate a trend towards hyperplasia.

Figure 7 summarizes these pattern observations. SdDM secretome appears to induce hypertrophy of visceral adipocytes. In this way, a lower number of adipocytes would harbor a larger triglyceride load (Figure 7, panel A and B, left). VdDM, in turn, seems to be inducing hyperplasia of subcutaneous adipocytes. Thus, a higher number of adipocytes hold a lower lipid load (Figure 7, Panel C and D, right). Visceral adipocytes, however, behave in an opposite way when differentiated with VdDM (Figure 7, Panel C and D, left).

### 3.3. Presence of Adipokines in the Secretomes

**Figure 8:**
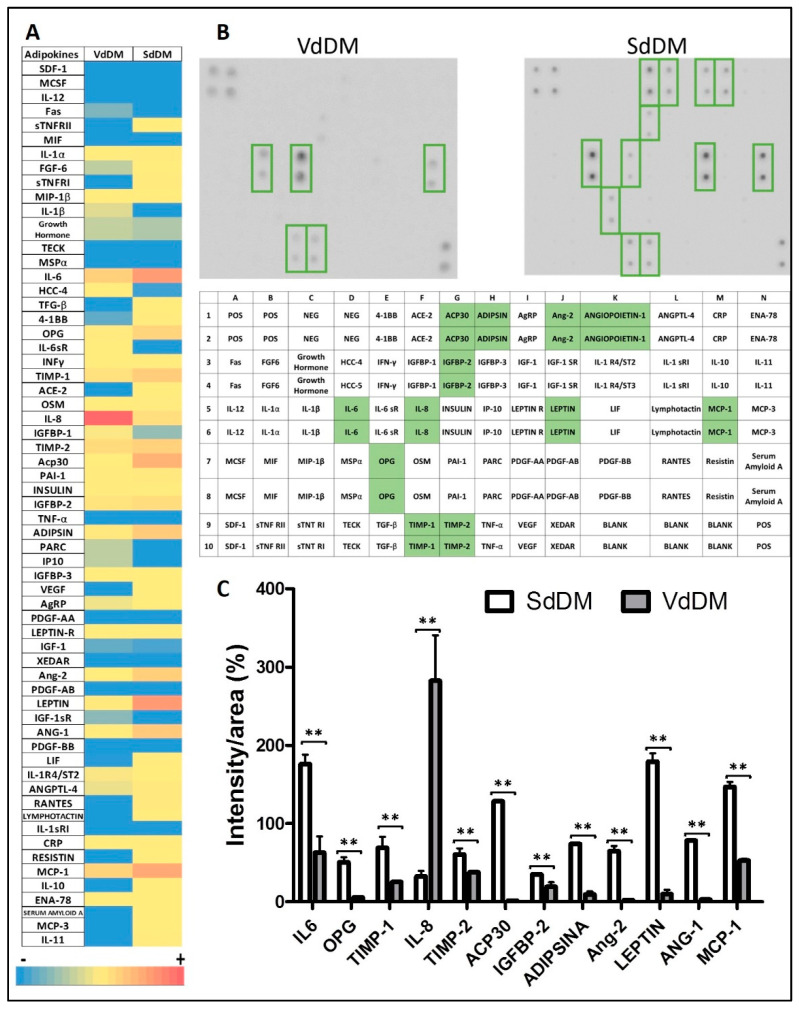
Expression of adipokines present in the secretomes (VdDM or SdDM). Panel A. Color-code heat maps of the density for each array, ranging from zero (blue) to maximum value (red), depicting the different adipokines in rows and the secretomes in columns. Panel B. Upper: array of the VdDM secretome; down: array of the SdDM secretome. Highly expressed adipokines are highlighted. Panel C. Semi-quantitative analysis using ImageJ of adipokines present in the secretomes. Each adipokine was normalized to the levels of POS control signals (which was set at 100% of intensity). Relative values of intensity for each adipokine are shown. Bars indicate mean ± SD of percentage intensity/area from the duplicate spots. Significant differences are represented as: ** adipokine comparisons between secretomes *p* < 0.01.

To assess the pathogenic potential of secretomes from DM2–PA differentiation, an antibody array kit, which includes 62 human adipokines related obesity, was used for each secretome. To visualize results and facilitate comparisons, we generated color-coded heat maps of the density for each array. As shown in Figure 8, panel A and B, twelve adipokines showed different intensities in the two arrays. The following were found: Adiponectin (Acp30), adipsin, leptin, specific inhibitors of metalloproteinases 1 and 2 (TIMP-1 and TIMP-2), insulin-like growth factor binding protein 2 (IGFBP-2), osteoprotegerin (OPG), angiopoietin-1 and 2 (ANG-1 and Ang-2, respectively), interleukine-6 and 8 (IL-6 and IL-8, respectively) and monocyte chemoattractant protein (MCP-1). Our results showed that all adipokines except IL-8, were increased in the SdDM respect to VdDM array. Among all the adipokines found, four are particularly noteworthy, Il-6, Acp30 and leptin in the SdDM and IL-8 in the VdDM array (Figure 8, panel C). The IL-8 value in the VdDM array was found to be approximately nine fold higher than that found in the SdDM array while that IL-6 in the SdDM array increased three fold respect to the value found in the VdDM array (Figure 8, panel C). On the other hand, the expression of Acp30 and leptin was very strong in SdDM while that minor values were obtained from VdDM array, even so a higher expression for leptin than for adiponectin was found in both secretomes. Of note, a low adiponectin/leptin ratio has been proposed as a marker of adipose tissue dysfunction [37].

## 4. Discussion

In the present study we investigated the effect on the cellular viability and accumulation of intracellular fat of healthy SAT and VAT mature adipocytes when differentiated in a diabesogenic microenvironment. We also studied the influence of CCT in these processes in an aim to broaden the existing knowledge on its beneficial nutritional effects.

We collected secretomes from differentiated DM2–PA of SAT or VAT origin to simulate diabesogenic microenvironments (SdDM and VdDM, respectively). These secretomes contain signals which can determine the growth of the adipocytes originated de novo [38]. Our present results suggest that the differentiation of PA is specific to each depot and it is determined by the microenvironment the PA are exposed to and by the differences inherent of PA of different depots. Specifically, we observed that, (1) SdDM did not affect neither cellular viability nor intracellular fat of the subcutaneous adipocytes; (2) SdDM secretome induced, during the differentiation of visceral adipocytes, a decrease in viability and an increase in intracellular fat; (3) VdDM secretome induced, during the differentiation of subcutaneous adipocytes, an increase in viability and a decrease in intracellular fat; and (4) the opposite, during the differentiation of visceral adipocytes. These observations suggest that the differentiation of SAT and VAT-derived adipocytes with SAT or VAT-derived secretomes, leads to certain patterns of hypertrophy/hyperplasia that merit discussion. SdDM secretome appears to induce hypertrophy of visceral adipocytes. This result implies that this secretome contains signals to block the differentiation of these PA, preventing the formation of mature adipocytes and, consequently it causes hypertrophy of mature adipocytes. VdDM, in turn, seems to be inducing hyperplasia of subcutaneous and hypertrophy of visceral adipocytes. The diminished expression of the mRNA for PPAR-γ in HV adipocytes differentiated with the VdDM secretome, would be in line with a hypothetic pattern of hypertrophy in the context of the secretory profile of this particular secretome, especially with that related to the expression of MCP-1, which has been reported to induce adipogenesis in the absence of PPAR-γ [39].

As stated above, SdDM activating differentiation HV PA, caused a decrease in viability, but exhibited a sharp rise in intracellular fat, following a hypertrophic growth pattern similar to the typical response of the VAT depot in obesogenic conditions. These results apparently disagree with the notion that the secretory profile of the SAT depot does not negatively influence metabolic homeostasis. However, our results suggest that the diabesogenic signals of SAT affect the expansion of VAT and possibly lead to a dysfunction of this depot. Visceral adiposity (reached mainly by adipocyte hypertrophy), compared to subcutaneous adiposity, is clearly linked to cardiometabolic disease and mortality [40]. Under this epidemiological belief we are probably underestimating the pathogenicity of SAT, especially the pathogenic action against VAT altering the activation of adipogenesis of this depot. This is relevant because, considering that SAT in pathological conditions like obesity, can interact negatively with the other depots, it might be possible to redirect the therapeutic strategies towards this depot. This is in line with some studies suggesting the importance of subcutaneous adiposity in metabolic abnormalities like visceral adiposity [41,42,43].

The secretory profile of VAT has been described as highly pro-pathological contributing to the pathogenic responses of SAT [4]. We expected that the secretome generated by visceral DM2 adipocytes (VdDM) will negatively influence the differentiation of healthy PA, especially subcutaneous PA because it is known that these PA are extremely sensible to changes in the extracellular medium [44]. However, our results suggest that VdDM leads to a hyperplasic expansion of subcutaneous adipocytes more alike a physiological de novo adipogenic process. This might occur in an attempt of SAT to fulfil its physiological buffer function. Since the pathogenic condition of this microenvironment should promote a hypertrophic expansion, the fact that a hyperplasic one prevails confirms the importance of the regulatory role of SAT. However, recent overfeeding studies have questioned the healthy role of hyperplasia suggesting that an increased population of small adipocytes is associated with impaired metabolic health outcomes [45,46]. Our results suggest that the metabolic consequences associated with visceral diabesity may be linked to hyperplastic expansion in SAT depot.

On the other hand, HV PA responded as expected, with a decrease in viability and an increase in intracellular fat. This could be indicative of a hypertrophic growth, in line with the well-known predisposition of VAT adipocytes to suffer hypertrophy [40]. In addition to adipose growth, adipocyte death is a vital component of adipose turnover. Indeed, an increased adipocyte death takes place in human obesity [47]. Our results showed that visceral adipocytes display a lower viability rate when differentiated with VdDM or SdDM. In contrast, viability of subcutaneous adipocytes was unaffected. This result suggests the existence of intrinsic differences in the differentiation process between human PA from the two depots when exposed to pathological conditions. In relation to the differentiation of HV PA, our findings are in line with several studies, which report that PA or mature adipocytes from VAT are more susceptible than those from SAT, to apoptotic stimuli, such as the tumor necrosis factor α (TNFα) [48]. It is possible that VdDM and SdDM contain proapoptotic signals that affect visceral adipocytes, either through an impairment of PA differentiation or by apoptosis of PA or of mature adipocytes.

The secretory profile analyzed for both secretomes supports the concept of “sick fat” [4], showing dysfunctional adipocytes especially from the microenvironment originated from DM2 subcutaneous adipocytes (as shown by the low adiponectin/leptin ratio) and a scenario of raised proinflammatory factors (Il-6 and IL-8) as potential mediators of diabesity. This suggests that the new adipocytes formed via diabesogenic adipogenesis may suffer an altered function, and thus directly contribute to endocrine dysfunction in AT, independently of an increased demand for fat storage.

Our results testing the effect of the solvent of CCT, DMSO, (the concentration of which, according to the literature, cannot be harmful to cells), corroborates the extreme sensitivity of HS PA. This cytotoxicity in response to DMSO did not occur in visceral adipocytes, highlighting additional differences between the inherent characteristics of both PA depots. The sensitivity of subcutaneous adipocytes to DMSO cytotoxicity was masked by the presence of CCT which exhibited a cytoprotective activity. It has been reported that CCT inactivates reactive oxygen species (ROS) and activates erythroid-derived nuclear factor-2 (Nfr2), a transcription factor involved in the expression of genes encoding for antioxidant enzymes [49]. It is likely that the antioxidant power of CCT is behind of the cytoprotection we observe in our experiments. It must be emphasized that these cytoprotective properties are evident when PA are differentiated with the non-pathological ADM. However, incubation with SdDM precluded the cytoprotective action of CCT in subcutaneous adipocytes while this was partially masked by VdDM. Obesogenic signals generate oxidative and inflammatory stress to different extents depending on the fat depots [38]. Our observations are possibly the result of the ability of CCT to compensate a probable stress exerted by our diabesogenic signals.

We have shown that CCT acts as an antiadipogenic compound. Other researchers have also reported the antiadipogenic effect of CCT, showing attenuation of accumulation of intracellular fat by CCT in visceral adipocytes of rats and mice [26]. Recently, our group has reported that CCT diminished intracellular fat during the differentiation of 3T3-L1 adipocytes by inhibiting CCAAT/enhancer-binding protein (C/EBPα) expression [50]. Saffron contains several bioactive compounds, of which crocins (crocetin esters) also exert antiadipogenic activity. Gu et al., reported that this effect of crocins occurred by activation of AMP-activated protein kinase (AMPK) [51], which is a fundamental sensor and modulator of metabolism. Rayalam et al., reported that the activation of AMPK inhibits differentiation of 3T3-L1 adipocytes, by blocking the expression of transcription factors, such as C/EBPα and PPARγ among others factors [52]. Considering that AMPK dysregulation is associated with obesity and DM2 and that CCT (which is the precursor of crocins) inhibits C/EBPα [50], it is likely that our results with CCT are caused by an AMPK-dependent C/EBPα inhibition.

In summary, our results show that exposure to diabesogenic microenvironments induces a greater disruption in the differentiation of visceral than in subcutaneous adipocytes. Both VdDM and SdDM secretomes increase cytotoxicity and provide with a proadipogenic environment during the differentiation of visceral adipocytes, while subcutaneous are resistant to these harmful environments. CCT restores the adipogenic process altered by the secretomes in a concentration-dependent manner, thus showing antiadipogenic activity (except for the differentiation of subcutaneous PA in the presence of VdDM). Besides, CCT exhibits cytoprotective effects during pathogenic PA differentiation and in non-pathological conditions. Further studies will have to be performed to evaluate the temporal expression of transcription factors involved in the process of adipogenesis, to analyze possible differences between diabesogenic and obesogenic secretomes to explain why not all obese people develop DM2 and finally, to dilucidate the pathways by which CCT exerts its beneficial effects.

Our results point to a role for CCT as a potential candidate to be included in pharmacological therapies aimed at reverting adipose tissue accumulation in diabesity. The therapeutic use of CCT is being investigated by various groups besides ourselves [53,54].

## 5. Conclusions

In conclusion, our results show that the diabesogenic microenvironment of both SAT and VAT depots provide a proadipogenic setting for healthy visceral PA, while subcutaneous PA exhibits resistance to this. We also found that the presence of CCT 10 μM in these pathogenic microenvironments acts as an antiadipogenic compound.

## Figures and Tables

**Table 1:** Conditions established and media used on the differentiation of healthy preadipocytes (PA).

Differentiation Of Healthy PA		
Control conditions	Protocols	Utilized Media
Control of non-differentiation (undifferentiated)	PA maintained with PGM2	PGM2
Control of differentiation	PA differentiated with ADM	ADM
Control effect of CCT	PA differentiated with ADM in presence of CCT	ADM + CCT 1 μM
		ADM + CCT 10 μM
Control effect of solvent of CCT	PA differentiated with ADM in presence of DMSO	ADM + DMSO 0.001%
Experimental conditions	Protocols	Utilized Media
Effect of Pathologic microenvironment	PA differentiated with Subcutaneous DM	SdDM
	PA differentiated with Visceral DM	VdDM
Effect of CCT in presence of pathologic microenvironment	PA differentiated with Subcutaneous DM in presence of CCT	SdDM + CCT 1 μM
		SdDM + CCT 10 μM
	PA differentiated with Visceral DM in presence of CCT	VdDM + CCT 1 μM
		VdDM + CCT 10 μM

CCT: crocetin. DMSO: dimethyl sulfoxide (CCT solvent). ADM: adipogenic differentiation medium. PGM2: PA growth medium. ADM + CCT: crocetin dissolved in ADM. ADM–DMSO: CCT solvent dissolved in ADM. SdDM: secretome from subcutaneous DM2–PA. SdDM + CCT: crocetin dissolved in the secretome from subcutaneous DM2–PA. VdDM: secretome from visceral DM2–PA. VdDM + CCT: crocetin dissolved in the secretome from visceral DM2–PA.

**Table 2:** Primers sequences.

Primers Sequences		
Name	Forward	Reverse
PPAR-γ	ATTCTCAGTGGAGACCGCCC	GACTCATGTCTGTCTCCGTC
18S	GAGGATGAGGTGGAACGTGT	TCTTCAGTCGCTCCAGGTCT

**Table 3:** Summary of viability results.

	Adipocytes from Healthy PA											
Viability (%)	Visceral						Subcutaneous					
	CCT 0 μM	*n*	CCT 1 μM	*n*	CCT 10 μM	*n*	CCT 0 μM	*n*	CCT 1 μM	*n*	CCT 10 μM	*n*
ADM	100 ± 7.5	4	74.2 ± 2.1	4	99.9 ± 3.1	2	100 ± 3.3	3	73.2 ± 1.4 ^##^	3	73.7 ± 2.0 ^##^	3
SdDM	64.3 ± 1.7 *^,§^	4	82.7 ± 1.5 ^§^	3	81.6 ± 1.2	3	106.3 ± 2.1 ^§^	4	33.4 ± 1.8 **^,††,b,#^	4	16.4 ± 0.6 ***^,†††,bbb,a^	2
VdDM	31.6 ± 1.0 **	4	55.1 ± 0.4 **^,b,#^	3	80.2 ± 2.4 ^††,a^	3	161.3 ± 0.1 ***	3	34.1 ± 1.5 **^,†††,b,#^	4	21.8 ± 1.5 ***^,†††,bb,#^	2
ADM–DMSO	84.0 ± 1.4	3					12.4 ± 0.4 ***	3				
UD	113.5 ± 4.7	2					117.8 ± 2.1	2				

Data obtained from the negative control of differentiation (UD), as well as statistical comparisons between the same type of adipocyte exposed to different microenvironments (§) are included. Values expressed as mean ± EEM, obtained on day 17 post-induction of differentiation. “n”: number of replicates averaged. CCT: crocetin. ADM: control differentiation medium. SdDM: secretome from differentiated subcutaneous DM2–PA. VdDM: secretome from differentiated visceral DM2–PA. ADM–DMSO: CCT solvent control. UD: undifferentiated PA, negative control of differentiation. Significant differences are represented as: *, **, *** compared to ADM, *p* < 0.05, *p* < 0.01 and *p* < 0.001, respectively. ††, ††† compared to SdDM or VdDM, *p* < 0.01, and *p* < 0.001, respectively. #, ## compared to DMSO, *p* < 0.05 and *p* < 0.01, respectively. “a” comparisons between CCT dose, *p* < 0.05. “b, bb, bbb” comparisons between VdDM or SdDM–CCT and ADM–CCT (same dose), *p* < 0.05, *p* < 0.01 and *p* < 0.001. “^§^” Comparisons between the effect of the different pathogenic secretomes over the viability of the same adipocyte type, *p* < 0.05.

**Table 4:** Summary of intracellular fat results.

	Adipocytes from Healthy PA											
Intracellular Fat (%)	Visceral						Subcutaneous					
	CCT 0 μM	*n*	CCT 1 μM	*n*	CCT 10 μM	*n*	CCT 0 μM	*n*	CCT 1 μM	*n*	CCT 10 μM	*n*
ADM	100 ± 0.5	4	107.5 ± 1.0	4	69.4 ± 0.6 *^,a,#^	2	100 ± 1.1	4	93.3 ± 1.5	4	52.0 ± 0.2 *^,a,#^	3
SdDM	195.0 ± 1.0 **^,§^	4	112.3 ± 2 ^†,§^	3	45.7 ± 1.1 **^,†††,aaa,b,§,#^	3	101.3 ± 1.1	4	74.1 ± 1.4	2	43.9 ± 0.8 **^,††,a,§,#^	3
VdDM	130,1 ± 1.5 *	4	147.9 ± 0.7 **^,b,#^	3	59.4 ± 1.5 *^,†,a,#^	3	93.1 ± 1.3	4	74.9 ± 2.8	3	78.9 ± 1.1 ^b^	3
ADM–DMSO	81.7 ± 0.7	3					79.6 ± 0.7	3				
UD	37.0 ± 0.3 ***	2					37.0 ± 0.3 ***	2				

Data obtained from the negative control of differentiation (UD), as well as statistical comparisons between the same type of adipocyte exposed to different microenvironments (§) are included. Values expressed as mean ± EEM, obtained on day 17 post-induction of differentiation. “*n*”: number of replicates averaged. CCT: crocetin. ADM: control differentiation medium. SdDM: secretome from differentiated subcutaneous DM2–PA. VdDM: secretome from differentiated visceral DM2–PA. ADM–DMSO: CCT solvent control. UD: undifferentiated PA, negative control of differentiation. Significant differences are represented as: *, **, *** compared to ADM, *p* < 0.05, *p* < 0.01 and *p* < 0.001, respectively. †, ††, ††† compared to SdDM or VdDM, *p* < 0.05, *p* < 0.01, and *p* < 0.001, respectively. # compared to DMSO, *p* < 0.05. “a, aaa”, comparisons between CCT dose, *p* < 0.05 and *p* < 0.001, respectively. “b” comparisons between VdDM or SdDM–CCT and ADM–CCT (same dose), *p* < 0.05. “§” Comparisons between the effect of the different pathogenic secretomes over the differentiation of the same adipocyte type, *p* < 0.05.

## Data Availability

The data presented in this study are available on request from the corresponding author.
